# Dissecting distinct proteolytic activities of FMDV L^pro^ implicates cleavage and degradation of RLR signaling proteins, not its deISGylase/DUB activity, in type I interferon suppression

**DOI:** 10.1371/journal.ppat.1008702

**Published:** 2020-07-15

**Authors:** Linda J. Visser, Chiara Aloise, Kirby N. Swatek, Gisselle N. Medina, Karin M. Olek, Huib H. Rabouw, Raoul J. de Groot, Martijn A. Langereis, Teresa de los Santos, David Komander, Tim Skern, Frank J. M. van Kuppeveld

**Affiliations:** 1 Virology Division, Department of Infectious Diseases and Immunology, Faculty of Veterinary Medicine, Utrecht University, The Netherlands; 2 Protein and Nucleic Acid Chemistry Division, Medical Research Council Laboratory of Molecular Biology, Cambridge, United Kingdom; 3 Department of Molecular Machines and Signaling, Max Planck Institute of Biochemistry, Martinsried, Germany; 4 United States Department of Agriculture, Agricultural Research Service, Foreign Animal Disease Research Unit, Plum Island Animal Disease Center, Orient, New York, United States of America; 5 Department of Medical Biochemistry, Max Perutz Labs, Vienna Biocenter, Medical University of Vienna, Vienna, Austria; 6 Ubiquitin Signaling Division, The Walter and Eliza Hall Institute of Medical Research, Parkville, Australia; 7 Department of Medical Biology, The University of Melbourne, Melbourne, Australia; Center for Infectious Disease Research, Medical College of Wisconsin, UNITED STATES

## Abstract

The type I interferon response is an important innate antiviral pathway. Recognition of viral RNA by RIG-I-like receptors (RLRs) activates a signaling cascade that leads to type I interferon (IFN-α/β) gene transcription. Multiple proteins in this signaling pathway (e.g. RIG-I, MDA5, MAVS, TBK1, IRF3) are regulated by (de)ubiquitination events. Most viruses have evolved mechanisms to counter this antiviral response. The leader protease (L^pro^) of foot-and-mouth-disease virus (FMDV) has been recognized to reduce IFN-α/β gene transcription; however, the exact mechanism is unknown. The proteolytic activity of L^pro^ is vital for releasing itself from the viral polyprotein and for cleaving and degrading specific host cell proteins, such as eIF4G and NF-κB. In addition, L^pro^ has been demonstrated to have deubiquitination/deISGylation activity. L^pro^’s deubiquitination/deISGylation activity and the cleavage/degradation of signaling proteins have both been postulated to be important for reduced IFN-α/β gene transcription. Here, we demonstrate that TBK1, the kinase that phosphorylates and activates the transcription factor IRF3, is cleaved by L^pro^ in FMDV-infected cells as well as in cells infected with a recombinant EMCV expressing L^pro^. *In vitro* cleavage experiments revealed that L^pro^ cleaves TBK1 at residues 692–694. We also observed cleavage of MAVS in HeLa cells infected with EMCV-L^pro^, but only observed decreasing levels of MAVS in FMDV-infected porcine LFPK αV*β*6 cells. We set out to dissect L^pro^’s ability to cleave RLR signaling proteins from its deubiquitination/deISGylation activity to determine their relative contributions to the reduction of IFN-α/β gene transcription. The introduction of specific mutations, of which several were based on the recently published structure of L^pro^ in complex with ISG15, allowed us to identify specific amino acid substitutions that separate the different proteolytic activities of L^pro^. Characterization of the effects of these mutations revealed that L^pro^’s ability to cleave RLR signaling proteins but not its deubiquitination/deISGylation activity correlates with the reduced IFN-β gene transcription.

## Author summary

Outbreaks of the picornavirus foot-and-mouth disease virus (FMDV) have significant consequences for animal health and product safety and place a major economic burden on the global livestock industry. Understanding how this notorious animal pathogen suppresses the antiviral type I interferon (IFN-α/β) response may help to develop countermeasures to control FMDV infections. FMDV suppresses the IFN-α/β response through the activity of its Leader protein (L^pro^), a protease that can cleave host cell proteins. L^pro^ was also shown to have deubiquitinase and deISGylase activity, raising the possibility that L^pro^ suppresses IFN-α/β by removing ubiquitin and/or ISG15, two posttranslational modifications that can regulate the activation, interactions and localization of (signaling) proteins. Here, we show that TBK1 and MAVS, two signaling proteins that are important for activation of IFN-α/β gene transcription, are cleaved by L^pro^. By generating L^pro^ mutants lacking either of these two activities, we demonstrate that L^pro^’s ability to cleave signaling proteins, but not its deubiquitination/deISGylase activity, correlates with suppression of IFN-β gene transcription.

## Introduction

A virally infected cell activates a plethora of antiviral responses. One of the best-known antiviral responses is the induction of type I interferons (IFN-α/β). Replication of the viral genome generates double-stranded RNA (dsRNA) replication intermediates that can be recognized by cytoplasmic RIG-I like receptors (RLRs). For example, picornaviruses, small (~30 nm) non-enveloped viruses with a positive-sense RNA genome, synthesize replication intermediates that are predominantly recognized by MDA5 [1–4]. Upon recognition of viral dsRNA, MDA5 interacts with MAVS, which subsequently activates TRAF3 and TBK1. TBK1 phosphorylates the transcription factors IRF3 and IRF7, resulting in their activation and dimerization. Simultaneously, TRAF3 interacts with the IKK complex to activate the transcription factor NF-κB. Upon activation, IRF3, IRF7 and NF-κB translocate to the nucleus, where they induce expression of IFN-α/β and other proinflammatory cytokines. Subsequent IFN-α/β signaling via the type I IFN receptor (IFNAR) and the JAK-STAT pathway induces the expression of hundreds of interferon stimulated genes (ISGs) (reviewed in [5,6]).

IFN-α/β gene transcription is extensively regulated by post-translational modification of RLRs and their downstream signaling proteins, including phosphorylation and ubiquitination. Ubiquitin is a 8.5 kDa protein that can be covalently linked through an ε-amino peptide linkage to lysine residues in target proteins. Within the RLR signaling pathway RIG-I, MAVS, TBK1, TRAF3, TRAF6 and IKKγ are ubiquitinated and this affects their molecular interactions, localization, stability, or activity (reviewed in [7,8]). Ubiquitination of RLR signaling proteins can both positively and negatively regulate the signaling pathway, which allows for rapid fine-tuning of the innate immune response against viral infection (reviewed in [7–9]). Consequently, many viruses encode enzymes with deubiquitinating (DUB) activity to manipulate the RLR signaling pathway and thereby suppress expression of IFN-α/β (reviewed in [9]).

In addition to ubiquitin, there are multiple ubiquitin-like modifiers, which can also be attached to target proteins. Of special interest is ISG15, an IFN-induced modifier of 17.5 kDa comprised of two ubiquitin-like domains in tandem. The exact antiviral properties of ISG15 are not yet fully understood (reviewed in [10,11]). Early work on ISG15 depended on mouse models and showed that expression of ISG15 protected mice from viral infection [12–15]. However, important biological and structural differences between ISG15 of murine and human origin have since been reported [16–19]. More recently, a picture is emerging that proteins are ISGylated co-translationally, explaining why predominantly viral proteins and ISGs are ISGylated upon infection in humans [20]. ISGylation of RLR signaling proteins has been reported, but the effect of these modifications on the outcome of the signaling pathway is still unclear ([21–24], reviewed in [11]). In addition, ISG15 has been reported to act as a cytokine [25,26].

IFN-α/β signals in autocrine and paracrine ways to induce a tissue-wide antiviral state, thereby limiting viral spread. To establish infection in their host, it is essential for viruses to suppress both the RLR signaling pathway and the downstream signaling of IFN-α/β. Affecting protein levels of important signaling molecules, either via cleaving them or inducing their degradation, is a strategy commonly used by viruses to suppress antiviral signaling [27–30]. One such example is the picornavirus foot-and-mouth disease virus (FMDV). FMDV is a member of the genus *Aphthovirus*, which also contains bovine rhinitis A and B viruses, and equine rhinitis A virus (ERAV). The genetic information on the FMDV RNA genome is translated as one polyprotein that is autocatalytically processed into the mature proteins, two of which have been shown to possess proteolytic activity and also been implicated in suppressing IFN-α/β induction (reviewed in [31]). The 3C^pro^, the protease that processes the majority of cleavage sites on the polyprotein, cleaves NF-κB essential modulator (NEMO), an adaptor protein that is essential to activate the NFκB and IRF signaling pathways [32].

The second protease on the polyprotein implicated in suppressing IFN-α/β induction is L^pro^, a papain-like cysteine protease located at the N-terminus of the polyprotein [33]. Once synthesized, L^pro^ immediately frees itself from the growing peptide chain by autocleavage at its own C-terminus. L^pro^ then efficiently cleaves the two isoforms of eIF (eukaryotic initiation factor) 4G to reduce protein synthesis from cellular mRNA [34] and suppresses the induction of IFN-α/β via several mechanisms. L^pro^ has been shown to induce the degradation of NF-κB subunit p65/RelA [35,36], and decrease the levels of IFN regulatory factor 3 (IRF3) and IRF7 [37]. Further, L^pro^ can also interact with ADNP, a negative regulator of transcription [38]. In addition to cleaving or degrading important signaling molecules, L^pro^ possesses deubiquitinase (DUB) activity which has been proposed to modulate RLR signaling [39]. A subsequent study demonstrated that L^pro^ should be predominantly regarded as a deISGylase rather than a DUB as biochemical evidence showed that L^pro^ has a 1000-fold higher affinity for ISG15 than for ubiquitin [40]. Structural studies and biochemical studies have shown separate substrate binding sites on L^pro^ for the viral polyprotein, the isoforms of eIF4G as well as for ubiquitin and ISG15 [40–42], suggesting that it may be possible to uncouple the activities of L^pro^ by the introduction of specific amino acid substitutions.

We therefore set out to uncouple the different activities of L^pro^ to discover whether L^pro^ suppresses RLR signaling through its deISGylase/DUB activity or through its ability to cleave and degrade multiple RLR signaling proteins. In this work, utilizing encephalomyocarditis virus (EMCV) expressing FMDV L^pro^ (EMCV-L^pro^), we identified MAVS and TBK1 as new L^pro^ substrates and determined the cleavage site in TBK1. By introducing specifically designed mutations into L^pro^, we further identified residues that are important for either the cleavage/degradation of RLR signaling proteins or for its deISGylase/DUB activity, thereby uncoupling the two catalytic activities of L^pro^. We demonstrate that cleavage/degradation of RLR signaling proteins, but not the deISGylase/DUB activity of L^pro^, correlates with suppressing IFN-α/β gene transcription.

## Results

### FMDV L^pro^ reduces IFN-β mRNA induction when introduced into a recombinant EMCV containing an inactive stress antagonist

**Fig 1:**
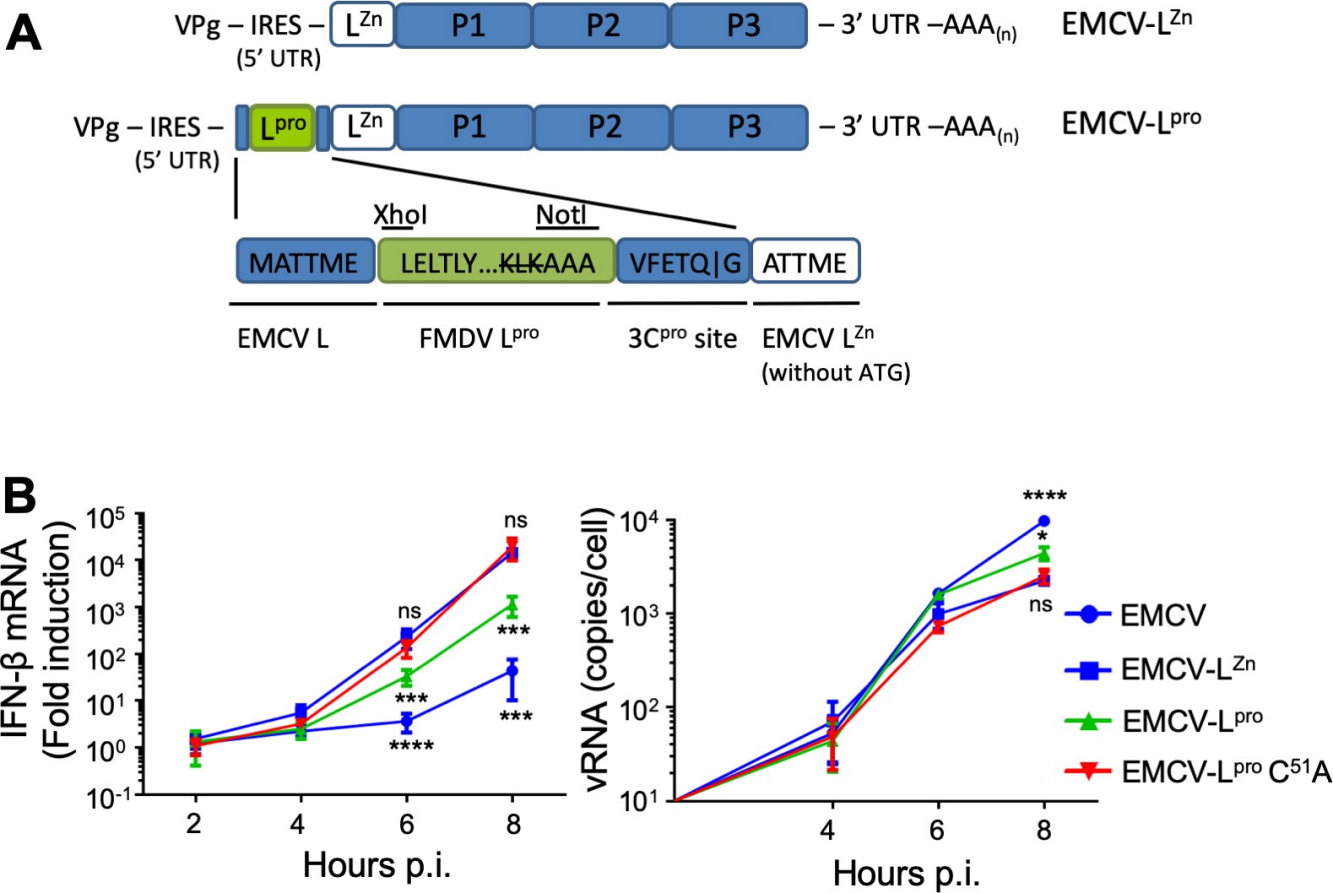
L^pro^ reduces IFN-β gene transcription when introduced into a recombinant EMCV containing an inactive stress antagonist. **(A)** Schematic representation of the recombinant EMCV-L^Zn^ and EMCV-L^pro^ viruses (the latter was described in detail in [45]). In EMCV-L^Zn^, L contains inactivating mutations in its Zn-finger domain (C19A/C22A) which abolishes its ability to suppress antiviral responses. To generate EMCV-L^pro^, the Lb^pro^ gene of FMDV O-strain was introduced at the 5’ end of the EMCV-L^Zn^ open reading frame. The L^pro^ cleavage site at its own C-terminus was mutated to AAA. Instead L^pro^ is released from this viral polyprotein via an EMCV 3C cleavage site. **(B)** Hela R19 cells were infected at MOI 10 with the indicated viruses and cells were lysed at 2,4,6,8 h pi. Total RNA was isolated and used for RT-qPCR analysis for IFN-β and actin mRNA, and EMCV vRNA. The IFN-β levels are depicted as a fold induction compared to levels in mock-infected cells, after correction for actin mRNA levels. The EMCV vRNA is depicted as a copy number per cell, calculated from a plasmid standard. Error bars depict the SD. One-way ANOVA with the Dunnet post hoc test was used to determine statistical significance compared to the results for EMCV-L^Zn^-infected cells (*, p<0.05; ***, p<0.001; ****, p<0.0001; ns, no significant difference).

To study the effects of L^pro^ on the induction of type I IFN in picornavirus-infected cells, we used two previously generated recombinant viruses; EMCV-L^Zn^, which contains inactivating mutations in the zinc-finger domain of the Leader (i.e. EMCV’s RLR signaling antagonist) [1,43,44], and EMCV-L^pro^, which was derived from EMCV-L^Zn^ and additionally encodes FMDV L^pro^ at the N-terminus of its polyprotein (Fig 1A) [45]. We also constructed a similar recombinant EMCV carrying a catalytically inactive L^pro^ (i.e. EMCV-L^pro^ C51A) [46]. To determine whether EMCV-L^pro^ can suppress IFN-β induction, we infected HeLa cells with EMCV, EMCV-L^Zn^, EMCV-L^pro^ or EMCV-L^pro^ C51A and determined the IFN-β mRNA levels over time via RT-qPCR analysis (Fig 1B). Consistent with previous studies [37,39,47], wt L^pro^, but not L^pro^ C51A, reduced the induction of IFN-β mRNA approximately 10-fold, indicating that the catalytic activity of L^pro^ is needed to suppress RLR signaling. In conclusion, the viruses that we generated (EMCV-L^pro^ and EMCV-L^pro^ C51A) accurately mimic the suppression of RLR signaling by L^pro^ as previously reported for FMDV-infected cells [47], providing us a model system to determine the mechanism via which L^pro^ suppresses type I IFN induction.

### L^pro^ cleaves the RLR signaling proteins MAVS and TBK1

**Fig 2:**
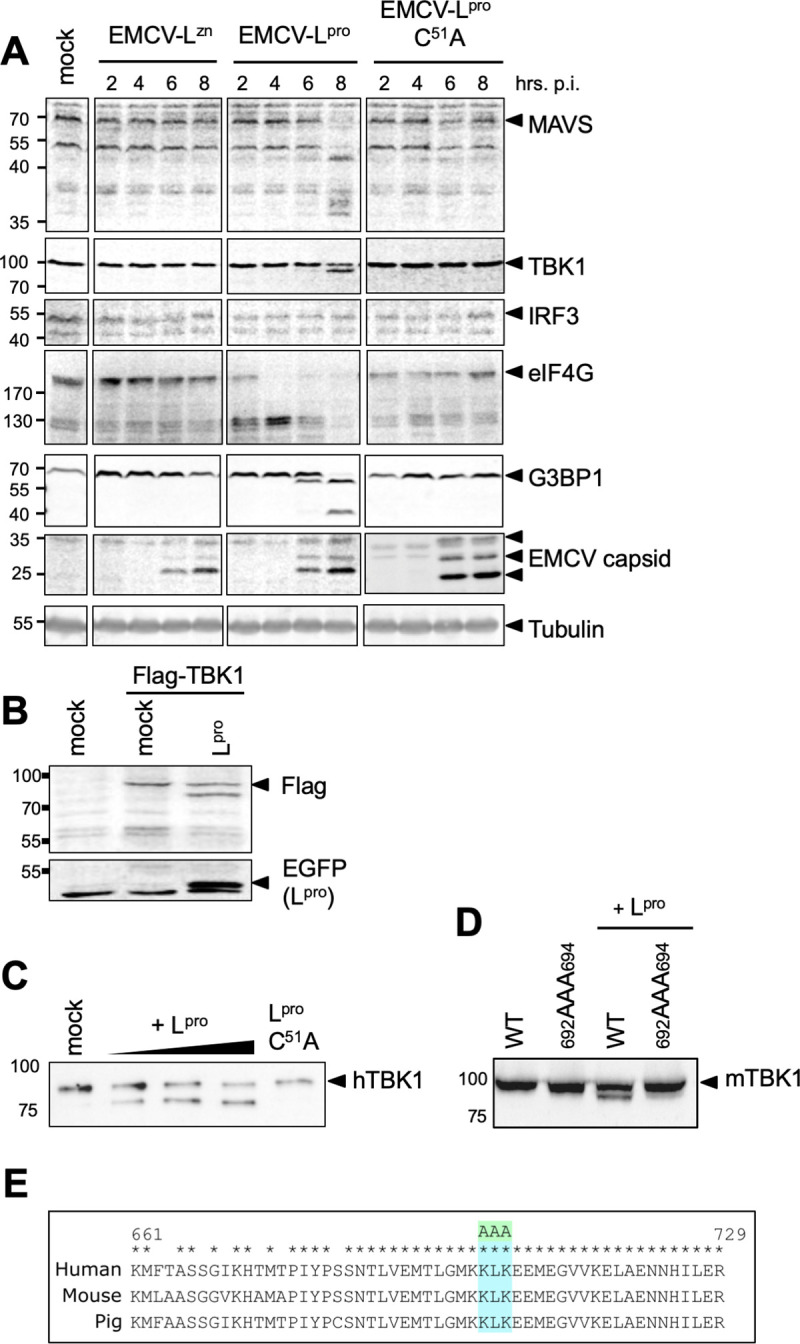
L^pro^ cleaves MAVS and TBK1. **(A)** HeLa R19 cells were infected at MOI 10 with the indicated viruses and lysed at 2,4,6,8 h pi. Cell lysates were subjected to Western Blot analysis for MAVS, TBK1, IRF3, eIF4GI, G3BP1, EMCV capsid proteins and tubulin. **(B)** HEK293T cells were transfected with 1 μg pcDNA-FLAG-TBK1 plasmid and 0.5 μg pIRES-GFP-L^pro^ plasmid. 16 h post transfection cells were lysed and lysates were subjected to Western Blot analysis for FLAG and GFP. **(C)** His-hTBK1 was incubated with 0–3 μg sL^pro^ or 3 μg sL^pro^ C51A for 2 h and reaction mixtures were subsequently subjected to Western Blot analysis for His. **(D)** HeLa OHIO cells were transfected with 7 μg pCS2-6Myc-mTBK1 or pCS2-6Myc-mTBK1_692AAA694_. 24 h post transfection, cells were lysed and incubated with 2 μg recombinant sL^pro^ for 2 h before being subjected to Western Blot analysis for HA. **(E)** Comparison of TBK1 amino acids 661–729 of human and mouse origin. Asterisks denote conserved residues. KLK at position 692–694 was mutated to AAA.

L^pro^ has been reported to degrade important signaling proteins such as the p65 subunit of NF-κB, IRF3 and IRF7 [35,37]. To determine whether L^pro^ targets additional RLR signaling proteins, we subjected cell lysates of HeLa cells infected with EMCV-L^Zn^, EMCV-L^pro^ or EMCV-L^pro^ C51A to Western Blot analysis for the signaling proteins MAVS, TBK1 and IRF3, as well as the known L^pro^-substrates eIF4G and G3BP1 (Fig 2A). EMCV capsid proteins and tubulin served as infection and loading controls, respectively. Infection with EMCV-L^pro^, but not EMCV-L^pro^ C51A, resulted in the rapid cleavage of eIF4G (from 4 hpi onwards) and the cleavage of G3BP1 (from 6 hpi onwards). We did not observe cleavage or degradation of IRF3 as was suggested by others [37]. In addition to these known cleavages, we observed cleavage of MAVS and TBK1 at 8 hpi. For MAVS, we observed multiple cleavage products ranging in apparent molecular weight from ~45 kDa to ~35 kDa. TBK1 cleavage resulted in a single cleavage product with an apparent molecular weight of ~90–95 kDa. We also attempted to detect MDA5 and investigate whether this dsRNA sensor is targeted by L^pro^. Unfortunately, the low levels of MDA5 prevented us from detecting the endogenous protein. MDA5 expression could be boosted by pretreatment with recombinant IFN-α2, but IFN-α2 pretreatment inhibited efficient EMCV infection, thereby interfering with the subsequent analysis.

We next focused our attention to identifying the cleavage site in TBK1. To this end, we overexpressed N-terminally FLAG-tagged TBK1 together with GFP-tagged L^pro^ and performed Western Blot analysis. As seen in Fig 2B, GFP-L^pro^ was able to cleave FLAG-TBK1. We observed an αFLAG-reactive cleavage product migrating at ~90–95 kDa, the same apparent molecular weight as the cleavage product we observed in EMCV-L^pro^ infected cells (Fig 2A), suggesting that L^pro^ cleaves TBK1 at its C-terminus. We also co-incubated recombinant His-TBK1 with increasing amounts of recombinantly expressed L^pro^ and L^pro^ C51A (Fig 2C). The *in vitro* incubation of His-TBK1 with wt L^pro^ also resulted in a ~90–95 kDa αHis-reactive cleavage product, confirming that L^pro^ cleaves TBK1 at its C-terminus and does not rely on other cellular factors. Incubation of His-TBK1 with catalytically inactive L^pro^ did not result in the formation of a cleavage product, confirming that the cleavage is dependent on L^pro^’s proteolytic activity. Subsequently, we showed that L^pro^ also cleaves TBK1 of murine origin (Fig 2D), which suggests that L^pro^ cleaves TBK1 in a conserved region. We identified residues _692_KLK_694_ –which localize at the very C-terminus of TBK1 and are well conserved between human, murine and porcine TBK1 –as a possible cleavage site (Fig 2E). Indeed, mutation of these residues prevented the cleavage of TBK1 by L^pro^ (Fig 2D), confirming these residues are the cleavage site.

Upon identifying the cleavage site in TBK1, we next investigated whether the cleavage of TBK1 by L^pro^ inhibits its function in the RLR signaling pathway. To this end, we generated cells in which the endogenous TBK1 gene is replaced with a TBK1 truncation mutant representative of the ~90–95 kDa cleavage product. We first generated HeLa TBK1 k.o. cells using CRISPR/cas9 technology and characterized the remaining RLR signaling capacity of these cells (S1A and S1B Fig). We found that depletion of TBK1 is not sufficient to fully impair RLR signaling, probably because of functional redundancy with IKKε (S1B Fig). TBK1 k.o. cells have a ~10-fold lower IFN-β induction upon infection with EMCV-L^Zn^, transfection of vRNA or upon overexpression of MAVS. As expected, IFN-β induction resulting from transfection of IRF3, which acts downstream of TBK1, was not affected in the TBK1 k.o. cells. Subsequently, we expressed full length TBK1 or TBK1 Δ35aa, which represents the N-terminal cleavage product, in TBK1 k.o. cells (S1C Fig) and determined whether expression of TBK1 and TBK1 Δ35aa restored IFN-β mRNA expression upon transfection of poly (I:C). Expression of full length TBK1 in TBK1 k.o. cells fully restored IFN-β mRNA induction (S1D Fig) and TBK1 Δ35aa was similarly efficient in this (S1E Fig), indicating that the L^pro^-generated N-terminal cleavage product is signaling competent.

### TBK1 is cleaved during aphthovirus infection

**Fig 3:**
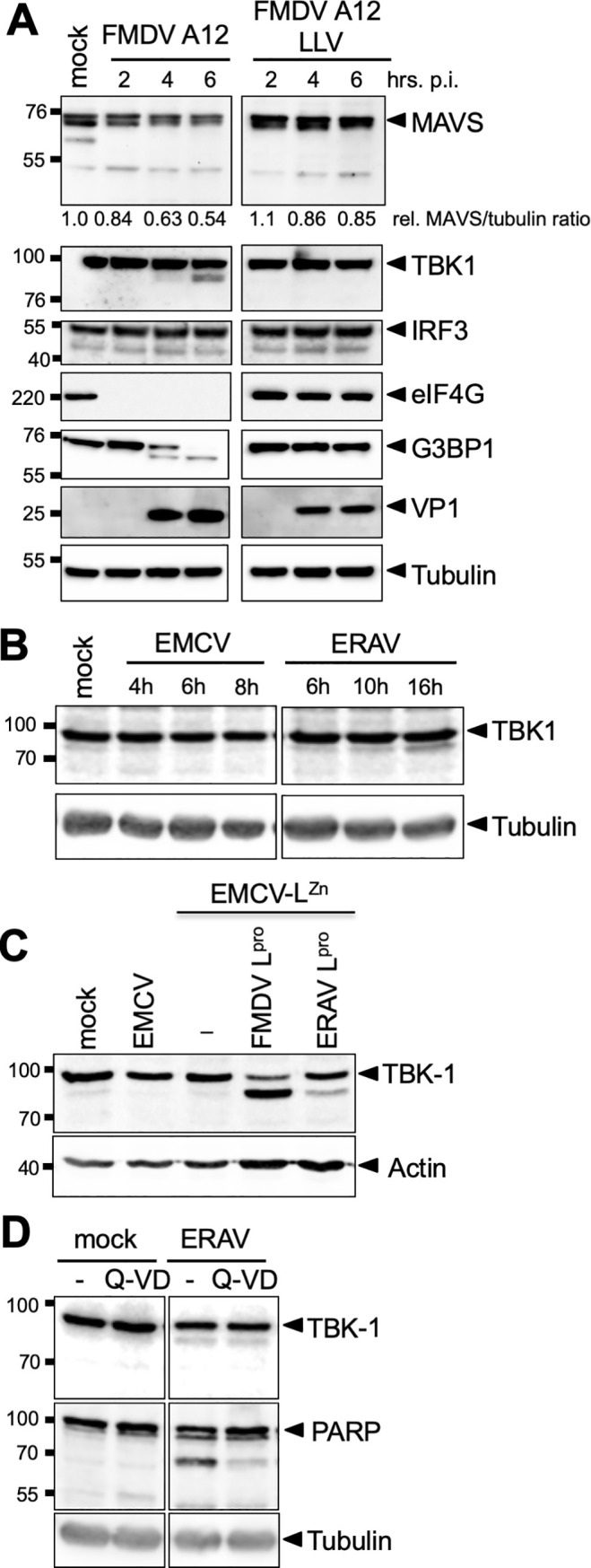
TBK1 is cleaved in aphthovirus infected cells. **(A)** LFPK αv*β*6 cells were infected with FMDV-A12 or leaderless FMDV-A12 (LLV) at MOI 10 and lysed at the indicated times postinfection. Lysates were subjected to Western blot analysis for MAVS, TBK1, IRF3, eIF4G, G3BP1, viral protein VP1 and tubulin. **(B)** HeLa R19 cells were infected with EMCV or ERAV at MOI 10 and lysed at indicated times post infection. Cell lysates were subjected to Western Blot analysis for TBK1 and tubulin. **(C)** HeLa R19 cells were infected at MOI 10 with the indicated viruses and lysed at 8 hpi. Cell lysates were subjected to Western Blot analysis for TBK1 and actin. **(D)** HeLa R19 cells were infected with ERAV at MOI 10. Subsequently, the cells were incubated for 16h in the presence or absence of 10 μM Q-VD, a pan-caspase inhibitor. Cell lysates were subjected to Western Blot analysis for TBK1, PARP and tubulin.

To investigate whether TBK1, MAVS and IRF3 are cleaved during FMDV infection, we infected porcine LFPK αv*β*6 cells with wt FMDV-A12 or FMDV-A12 lacking L^pro^ (leaderless virus, A12-LLV). Western Blot analysis revealed cleavage of TBK1, but not of MAVS or IRF3, during wt FMDV-A12 infection (Fig 3A). TBK1 cleavage was observed from 4 hpi onwards upon infection with wt FMDV, but not upon infection with leaderless FMDV. The cleavage product had an apparent molecular weight of ~90–95 kDa, consistent with our previous observations of the size of this cleavage product. Although a MAVS cleavage product was not detected during FMDV infection, densitometry analysis revealed a strong and progressive decrease in the relative ratio of MAVS/tubulin from 2–6 hpi post infection with wt FMDV compared to mock-infected cells, whereas only a small decrease was detected in leaderless-infected cells (MAVS/tubulin ratio is indicated in Fig 3A). This suggests that expression of L^pro^ induces degradation of MAVS, also in FMDV-infected cells. Consistent with our observations in EMCV-L^pro^ infected cells, we did not observe a decrease in IRF3 signal in FMDV-infected cells.

To investigate whether the cleavage of TBK1 is conserved amongst aphthoviruses, we infected cells with ERAV, the closest relative of FMDV. Infection with ERAV, but not EMCV, resulted in the cleavage of TBK1 (Fig 3B). However, the cleavage product was less prominent than for EMCV-L^pro^, suggesting that cleavage was inefficient or infection was delayed. Notably, in our HeLa cells, ERAV displayed a replicative cycle of ~16 h. This is considerably slower than FMDV, which replicates in 6–8 hours. To study TBK1 cleavage by the two different L^pro^’s irrespective of variation in viral replication kinetics, we infected cells with EMCV-L^pro^ or an EMCV expressing ERAV L^pro^ (EMCV-ERAV L^pro^), for which we previously determined the replication kinetics to be similar [45]. Both viral proteases cleaved TBK1 resulting in a ~90–95 kDa cleavage product (Fig 3C). FMDV L^pro^ cleaved TBK1 more efficiently than ERAV L^pro^, consistent with the results observed during infection with FMDV or ERAV (Fig 3A and 3B). Notwithstanding the differences between ERAV L^pro^ and FMDV L^pro^, our data demonstrate that the ability to cleave TBK1 is conserved amongst these two aphthoviruses.

We also investigated the effect of the pan-caspase inhibitor Q-VD-PH (Q-VD) on TBK1 cleavage in ERAV-infected cells (Fig 3D). While addition of Q-VD decreased the cleavage of known caspase substrate PARP, the cleavage of TBK1 was unaffected. Collectively, these results demonstrate that L^pro^ directly cleaves TBK1 and that this activity is conserved amongst aphthoviruses.

### Construction of L^pro^ mutants to uncouple cleavage/degradation of RLR signaling proteins from its deISGylase/DUB activity

**Fig 4:**
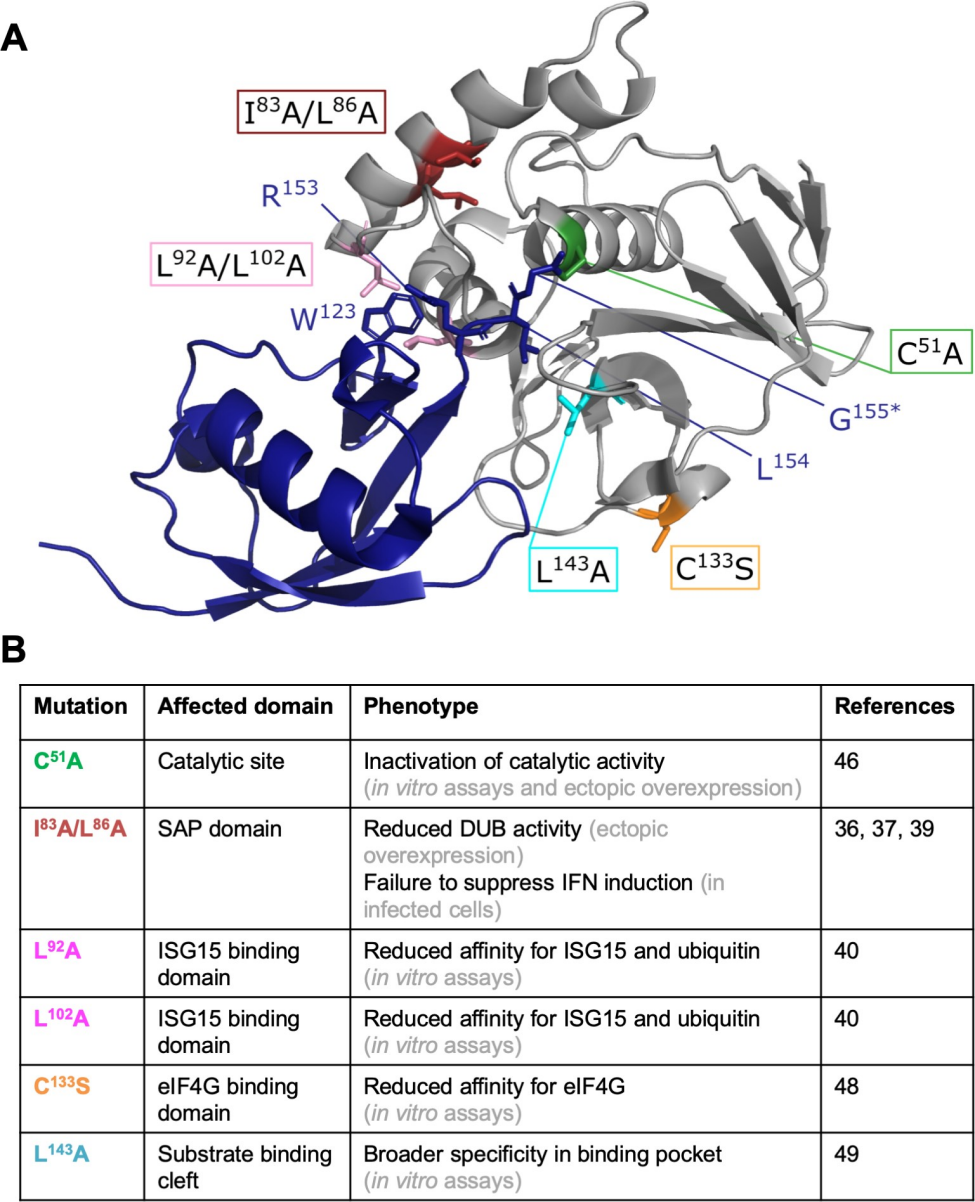
Visualization and summary of the L^pro^ mutants used in this study. **(A)** Standard cartoon view of L^pro^ (grey) bound to ISG15 (blue) (PDB: 6FFA). L^pro^ L92 and L102 interact with ISG15 W123, this positions ISG15’s C-terminus (R153, L154 and G155) in the substrate binding cleft of L^pro^. *In this structure a C-terminal propargyl warhead replaced G155 [40]. Residues which upon mutation were reported to affect L^pro^’s structure or function are shown as colored sticks. Green: C51A inactivates L^pro^’s catalytic activity; red: I83A or L86A reduce the DUB activity and IFN induction; pink: L92A and L102A reduce affinity for ISG15; orange: C133S reduces affinity for eIF4G; aquamarine: L143A predicted to open substrate binding pocket. Drawings were generated using PyMOL. **(B)** Overview of the reported effects of the introduced mutations in L^pro^ on the various proteolytic activities of L^pro^, this includes references and the category of the underlying experimental evidence.

L^pro^ also possesses deISGylase and—to a lesser extent—DUB activity [39,40,42], and this latter activity was previously suggested to be important for L^pro^’s ability to suppress RLR signaling [39]. To investigate how L^pro^ reduces the induction of IFN-β gene transcription, we set out to uncouple these two abilities of L^pro^. To this end, we introduced previously described mutations in the chimeric EMCV-L^pro^ and determined whether these mutations affect the deISGylase/DUB activity of L^pro^ and/or its ability to cleave/degrade RLR signaling proteins. A mutation in the SAP domain (I83A/L86A), was previously reported to abolish the ability of L^pro^ to suppress type I IFN expression, to degrade signaling proteins (i.e. NF-κB p65, IRF3 and IRF7), and to disrupt its DUB activity [36,37,39], and was therefore included in our screening. Analysis of the crystal structure of L^pro^ bound to ISG15 suggested that L^pro^ residues L92, P99 and L102 are important for ISG15 binding [40]. Fig 4A shows the structure of L^pro^ (grey) in complex with ISG15 (blue) and indicates the residues of ISG15 (W123 and R153, L154, G155) that interact with L^pro^. Mutation of L92, P99 or L102 in L^pro^ reduced its affinity for ISG15 and impaired its deISGylase activity, without affecting eIF4G cleavage [40]. Homology modeling showed that ISG15 and ubiquitin interact with the same surfaces of L^pro^ [40], suggesting that L^pro^’s deISGylase activity also reflects its DUB activity. Two of these mutations (L92A and L102A) were introduced in EMCV-L^pro^. In addition, we introduced the mutations L143A and C133S. Mutation C133S was reported to reduce the affinity of L^pro^ for eIF4G [48] whereas mutation of to L143 to alanine with its shorter side-chain rescued polyprotein processing in the context of the additional mutation L200F [49]. Based on the structure of L^pro^, mutation L143A has been predicted to open up the catalytic pocket. Fig 4 shows the locations of the residues that are mutated in this study (Fig 4A) and summarizes the reported effects of these mutations on L^pro^’s various proteolytic activities (Fig 4B).

### Effect of L^pro^ mutations on cleavage and/or degradation of RLR signaling proteins

**Fig 5:**
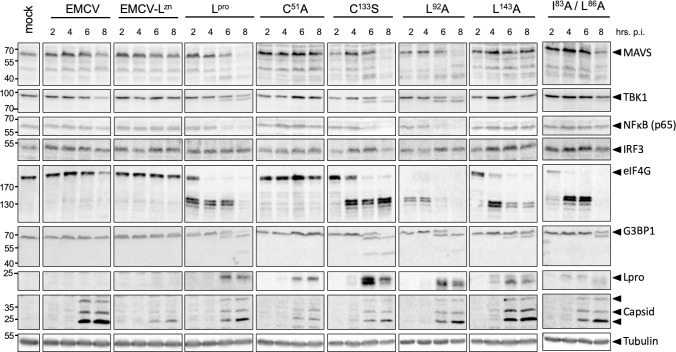
Mutation of L143 or SAP domain strongly reduces cleavage and/or degradation of RLR signaling proteins. HeLa R19 cells were infected at MOI 10 with the indicated viruses and lysed at 2, 4, 6, 8 h pi. Cell lysates were subjected to Western Blot analysis for MAVS, TBK1, NF-κB p65, IRF3, eIF4G, G3BP1, L^pro^, EMCV capsid proteins and tubulin.

**Fig 6:**
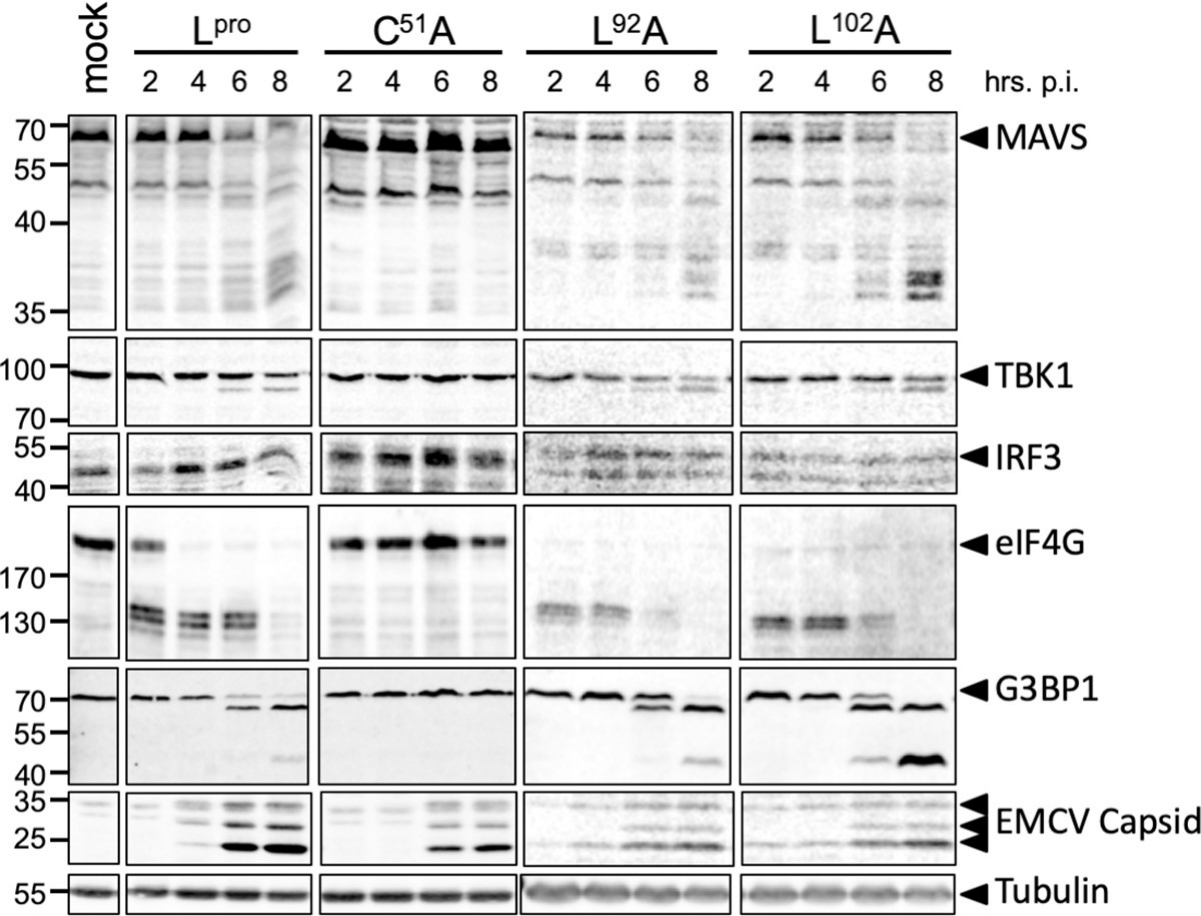
Neither mutation of L92 or L102 affects the cleavage and/or degradation of RLR signaling proteins. HeLa R19 cells were infected at MOI 10 with the indicated viruses and lysed at 2, 4, 6, 8 hpi. Cell lysates were subjected to Western Blot analysis for MAVS, TBK1, NF-κB p65, IRF3, eIF4G, G3BP1, L^pro^, EMCV capsid proteins and tubulin.

First, we determined the effect of the introduced mutations on L^pro^’s ability to cleave or degrade RLR signaling proteins. We infected Hela cells with EMCV, EMCV-L^Zn^ or the different EMCV-L^pro^ carrying the described mutations, lysed the cells at the indicated timepoints and subjected the lysates to Western Blot analysis for MAVS, TBK1, NF-κB subunit p65, IRF3, eIF4G and G3BP1, as well as L^pro^ and EMCV capsid proteins (Fig 5). Our data show that infection with the different L^pro^ mutant viruses resulted in L^pro^ expression and accumulation of EMCV capsid proteins from 6 hpi onwards, which is indicative of efficient infection. Interestingly, the introduced mutations in L^pro^ had different effects on the cleavage or degradation of RLR signaling proteins. Upon infection with EMCV-L^pro^ C133S, we observed a ~2 hr delay in eIF4G cleavage, consistent with a previous report [48]. This mutation did not affect the cleavage/degradation of the various RLR signaling proteins. Mutation of L92 or L102 has been reported to reduce the activity of L^pro^ towards ISG15, without affecting eIF4G cleavage [40]. Indeed, infection with EMCV-L^pro^ L92A, resulted in cleavage of eIF4G, as well as all other L^pro^ substrates (i.e. MAVS, TBK1, NF-κB p65 and G3BP1) (Fig 5). Infection with both EMCV-L^pro^ L92A and EMCV-L^pro^ L102A resulted in efficient cleavage of RLR signaling proteins, confirming that these two L^pro^ mutants have the same proteolytic profiles (Fig 6). Of note, the cleavage of eIF4G appears to occur faster by L^pro^ L92A and L102A compared to wt L^pro^, although a more in-depth analysis is necessary to confirm a true difference in eIF4G cleavage kinetics. Importantly, both mutations allow us to separate the deISGylase/DUB activity of L^pro^ from its ability to cleave RLR signaling proteins. Serendipitously, we observed that L^pro^ carrying mutation L143A was strongly impaired in degrading NF-κB p65 and cleaving MAVS and TBK1, while cleavage of G3BP1 and eIF4G was delayed but could be observed clearly at later timepoints (eIF4G cleavage is ~2 hr delayed, comparable to the delay observed for L^pro^ C133S). Mutation of L^pro^’s SAP domain (I83A/L86A), which was previously shown to abolish degradation of NF-κB p65 and DUB activity as well as to impair L^pro^’s ability to reduce IFN-β mRNA expression [36,39], also affected L^pro^’s ability to cleave MAVS and TBK1. Overall, our data demonstrate that the mutations have differential effects on the cleavage/degradation of RLR signaling proteins. Importantly, we demonstrate that L^pro^ residues L92 and L102, which are essential for its deISGylase/DUB activity, are not essential for is ability to cleave/degrade RLR signaling proteins, indicating that the two different catalytic activities of L^pro^ can be uncoupled.

### Effect of L^pro^ mutations on DUB and deISGylase activities

**Fig 7:**
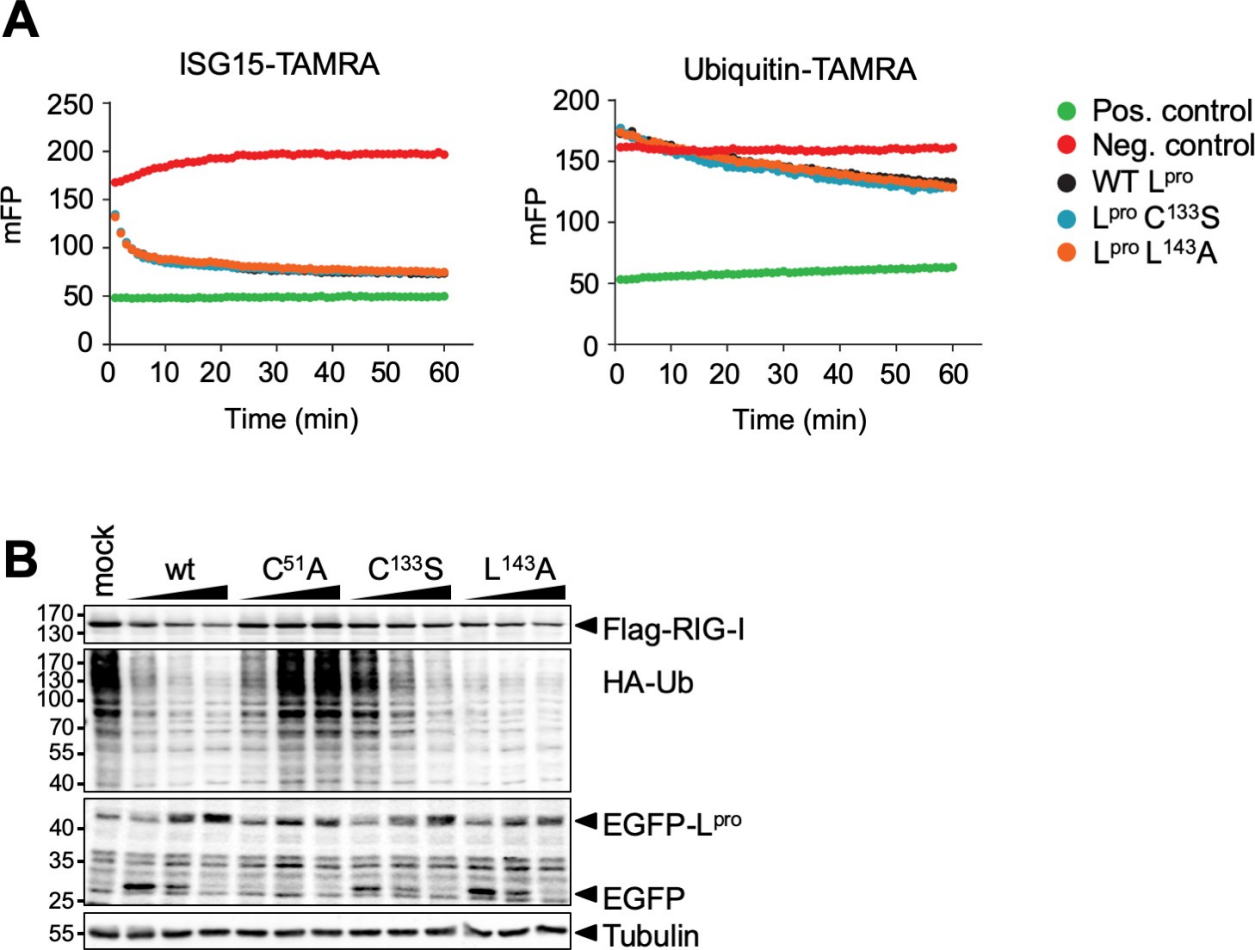
Mutation of L143 or C133S does not affect DUB or deISGylase activities. **(A)** Ubiquitin-TAMRA or ISG15-TAMRA (substrates) were incubated with 10 μM or 100 nM L^pro^ (wt or indicated mutant), respectively. The change in fluorescent polarization (mFP) was determined over 60 min, with readings recorded every 60 sec. The average trace of assays performed in technical triplicate is shown. Substrate only and KG-TAMRA peptide served as respective negative and positive controls. **(B)** HEK293T cells were transfected with 0.5 μg pcDNA-FLAG-RIG-I, 0.5 μg pcDNA-HA-ubiquitin and 0.1–0.5 μg pIRES-GFP-L^pro^. 16 h post transfection, cells were lysed and lysates subjected to Western Blot analysis for FLAG, HA, GFP and tubulin.

It has been previously reported that mutations L92A, L102A and I83A/L96A affect L^pro^’s deISGylase/DUB activity [39,40]. To determine the effect of mutations L143A or C133S on these activities, mutant L^pro^’s were expressed and purified from *E*. *coli* and *in vitro* catalytic activities towards ubiquitin-TAMRA and ISG15-TAMRA were measured (Fig 7A). L^pro^ C133S and L143A displayed wt-like activity towards ISG15 and ubiquitin. We next determined DUB activity of wt L^pro^, L^pro^ C51A, C133S and L143A in cells. To this end, we transfected HEK-293T cells with a combination of HA-ubiquitin, FLAG-RIG-I and increasing amounts of GFP-L^pro^ encoding plasmids (0.1, 0.3 and 0.5 μg), and visualized HA-tagged ubiquitinated proteins by Western Blot analysis (Fig 7B). FLAG-RIG-I, which was included to monitor the effects of L^pro^-induced translational shut-off on the overexpressed proteins, was clearly detectable even at the highest level of L^pro^ (i.e. transfection of 0.5 μg of L^pro^ plasmid), indicating that L^pro^-induced translational shut-off did not significantly reduce the protein expression from the transfected plasmids. Both wt L^pro^ as well as the C133S and L143A mutants displayed DUB activity upon overexpression in cells, as indicated by the reduction of HA-tagged ubiquitinated proteins upon increasing L^pro^ levels. Notably, we observed ubiquitinated proteins upon transfection of low amount of L^pro^ C133S plasmid (0.1 μg), although our *in vitro* data (Fig 7A) indicated that mutation C133S does not reduce the activity for ubiquitin. As the *in vitro* data are much more quantitative in nature, we consider the relatively decreased DUB activity of L^pro^ C133S in comparison to L^pro^ wt or mutant L143A as observed in Fig 7B the result of variations in expression of the transfected plasmids.

**Fig 8:**
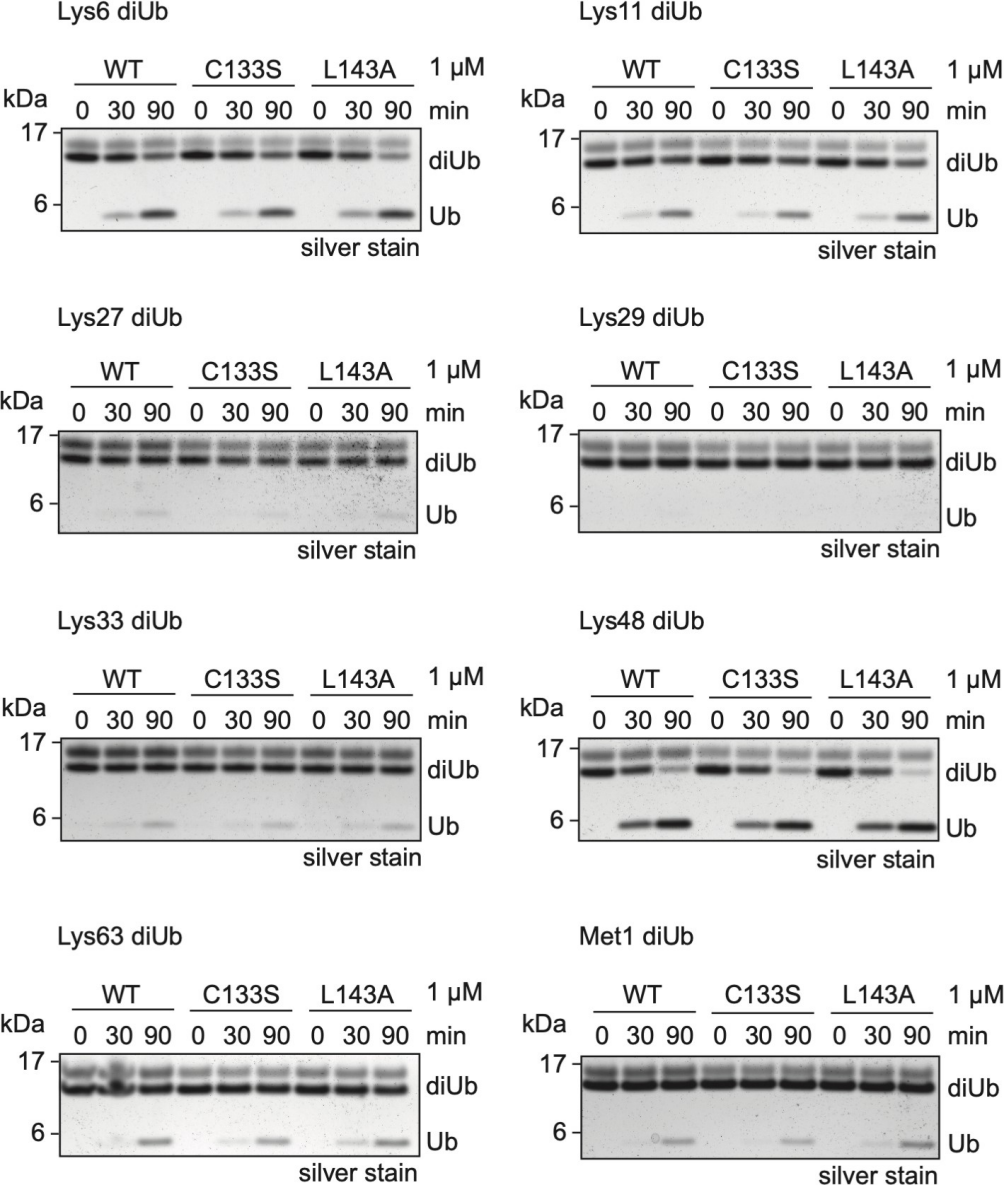
Mutation of L143 or C133 does not affect activity towards differently linked ubiquitin. Wild-type L^pro^ and the L^pro^ mutants (C133S, L143A) were incubated with each of the eight di-ubiquitin chain types for the indicated times, separated by SDS-PAGE, and visualized by silver-staining. The concentration of L^pro^ used in the cleavage assays is indicated.

Thus far, our data showed that mutations C133S and L143A did not affect L^pro^’s DUB activity towards a mono-ubiquitin fused to a fluorescent TAMRA molecule. To exclude the possibility that mutations C133S and L143A affect L^pro^’s DUB activity towards other substrates, we determined its ability to cleave differently-linked di-ubiquitin molecules. Co-incubation of L^pro^ with di-ubiquitin molecules of different linkages indicated that L^pro^ preferentially targets K6- and K48-linked ubiquitin chains, displayed some activity towards K11-, K33-, K63-, and M1-linked chains, but at this enzyme concentration and incubation times has no activity towards K27- or K29-linked ubiquitin chains (Fig 8). Neither mutation L143A nor C133S affected the ability of L^pro^ to cleave the di-ubiquitin molecules. Overall, our analysis shows that L^pro^ carrying mutation L143A or C133S have wt-like deISGylase and DUB activity. Importantly, as mutation L143A impairs L^pro^’s ability to cleave/degrade RLR signaling proteins (Fig 5), but does not affect its deISGylase/DUB activity, introduction of this mutation also allows us to make a distinction between the two proteolytic activities of L^pro^.

### L^pro^’s ability to reduce IFN-β mRNA levels correlates with its ability to cleave/degrade RLR signaling proteins, not with its deISGylase/DUB activity

**Fig 9:**
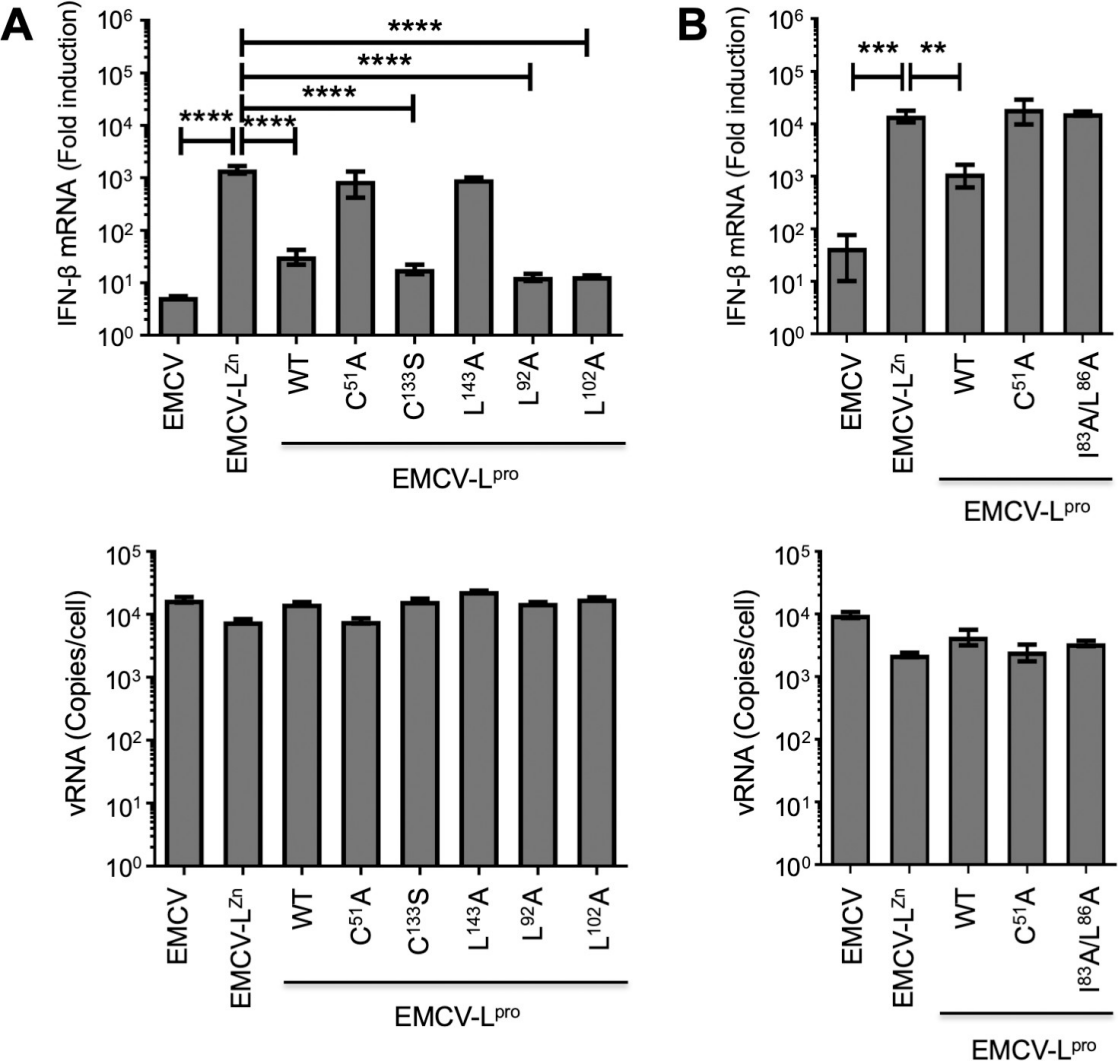
Reduction in IFN-β gene transcription correlates with cleavage and/or degradation of RLR signaling proteins. **(A+B)** Hela R19 cells were infected at MOI 10 with the indicated viruses and cells were lysed at 8 hpi. Total RNA was isolated and used for RT-qPCR analysis for IFN-β and actin mRNA (upper panels), and EMCV vRNA (lower panels). The IFN-β levels are depicted as a fold induction compared to levels in mock-infected cells, after correction for actin mRNA levels. The EMCV vRNA is depicted as a copy number per cell, calculated from a plasmid standard. Error bars represent the SD. One-way ANOVA with the Dunnet post hoc test was used to determine statistical significance compared to the results for EMCV-L^Zn^-infected cells (**, p<0.01; ***, p<0.001; ****, p<0.0001).

**Fig 10:**
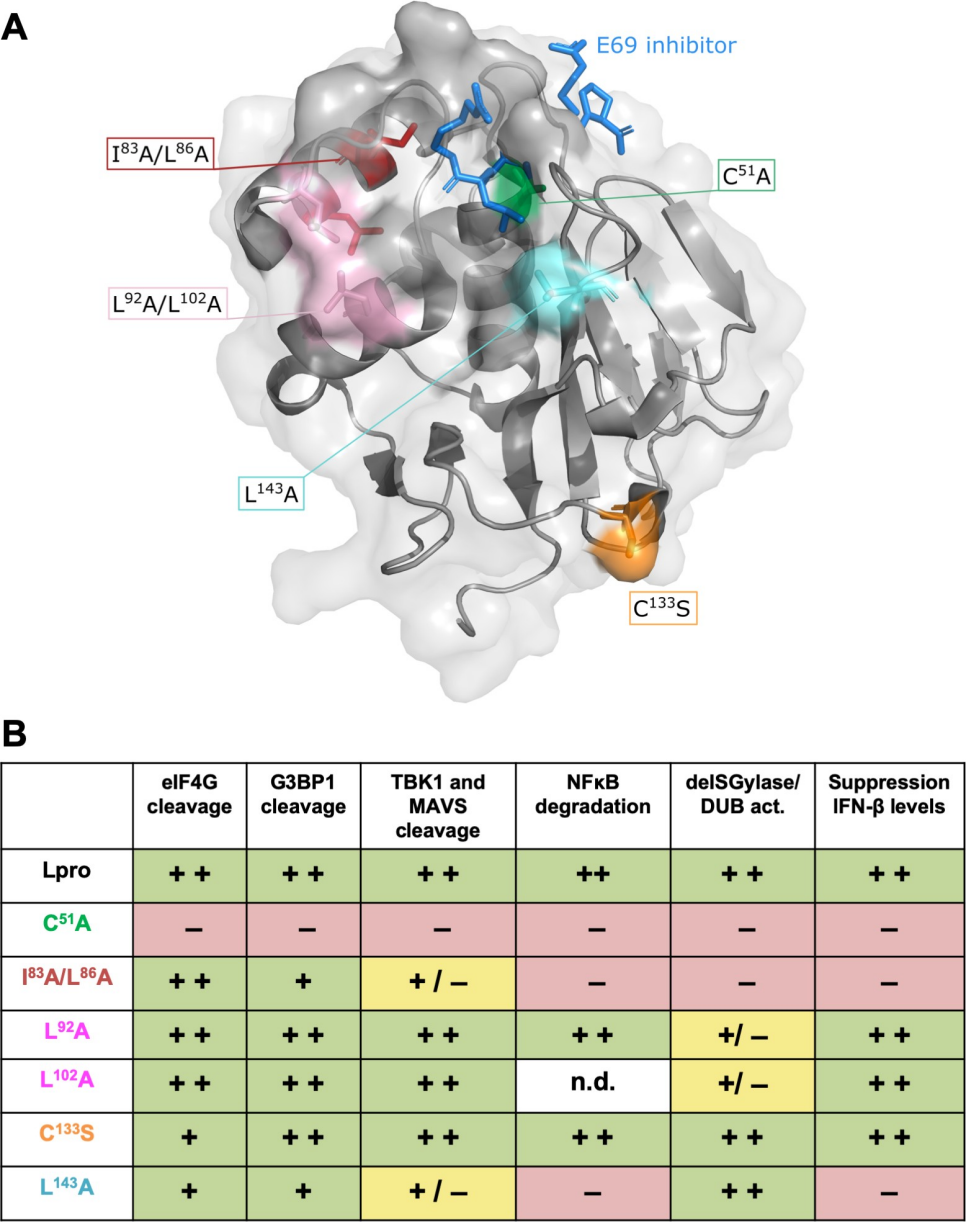
Overview of the effects of L^pro^ mutations on the different proteolytic activities of L^pro^ as well as reduction in IFN-β gene transcription. **(A)** Standard cartoon view with transparent surface of L^pro^ bound to E69 inhibitor shown as blue sticks (PDB: 4QBB). Residues which upon mutation were reported to affect L^pro^’s structure or function are shown as colored sticks. Green: C51A inactivates L^pro^’s catalytic activity; red: I83A or L86A reduce the DUB activity and IFN induction; pink: L92A and L102A reduce affinity for ISG15; orange: C133S reduces affinity for eIF4G; aquamarine: L143A predicted to open substrate binding pocket. Drawings were generated using PyMol. **(B)** Overview of the effects of introduced mutations on cleavage and/or degradation of host proteins, deISGylase/DUB activity, and their ability to reduce IFN-β gene transcription. Coloring of mutations is consistent with panel A. The activities of the mutations have been scored + +, +, + /–, or–according to the following criteria. + +, activity is similar to wt L^pro^; +, moderately reduced activity; + /–, severely impaired activity;–, no activity.

Having characterized how the different mutations in L^pro^ affect its deISGylase/DUB activity or the cleavage/degradation of RLR proteins, we next set out to determine which mutants reduce the induction of IFN-β mRNA. We infected HeLa cells with EMCV, EMCV-L^Zn^ or the different EMCV-L^pro^ mutants and determined the IFN-β mRNA and EMCV vRNA levels over time via RT-qPCR analysis (Fig 9). Wildtype L^pro^ as well as L^pro^ C133S, L92A and L102A consistently reduced the induction of IFN-β mRNA, while L^pro^ C51A and L143A were unable to do so (Fig 9A). The SAP domain mutant (EMCV-L^pro^ I83A/L86A) also failed to suppress IFN-β mRNA levels (Fig 9B), which is in agreement with observations in FMDV-infected cells [36,39]. Notably, infection with EMCV L^pro^ L143A, which displayed wt deISGylase/DUB activity but is strongly impaired in its ability to cleave/degrade RLR signaling proteins MAVS, TBK1 and NFκB p65, failed to suppress the induction of IFN-β mRNA. In contrast, L^pro^ L92A and L102A,which are strongly impaired in their deISGylase/DUB activity [40] but not in their ability to cleave/degrade RLR signaling proteins, reduced IFN-β mRNA levels. These combined observations (summarized in Fig 10) demonstrate that cleavage/degradation of RLR signaling proteins, but not the deISGylase/DUB activity of L^pro^, correlate with suppressing IFN-α/β gene transcription. Notably, L^pro^ C133S reduced the induction of IFN-β mRNA despite a 2h delay in eIF4G cleavage, indicating that the rapid eIF4G cleavage and the subsequent translational shut-off is not sufficient to suppress RLR signaling.

## Discussion

FMDV suppresses IFN-α/β both at the mRNA and at the protein level [47], but the molecular mechanism underlying the reduced induction of IFN-α/β gene transcription (RLR signaling) is poorly understood. Both the DUB activity of L^pro^ as well as its ability to cleave/degrade RLR signaling proteins have been implicated in the suppression of RLR signaling [35,37,39]. In this study, we identified MAVS and TBK1 as novel L^pro^ substrates and mapped the cleavage site in TBK1. Moreover, by introducing specific mutations we were able to separate L^pro^’s deISGylase/DUB activity from its ability to target RLR signaling proteins. Using L^pro^ carrying either of these uncoupling mutations, we demonstrated that the cleavage/degradation of RLR signaling proteins, not the deISGylase/DUB activity, correlates with the ability to reduce IFN-β gene transcription. Collectively, our data strongly suggest that the ability of L^pro^ to cleave/degrade RLR signaling proteins is needed to reduce the IFN-β mRNA levels.

We identified TBK1 as a new L^pro^ substrate and identified the cleavage site. We observed cleavage of TBK1 both in HeLa cells infected with EMCV-L^pro^ and in FMDV-infected LFPK cells, and we demonstrated that the L^pro^ cleavage site is located towards the C terminus of TBK1, more specifically in the coiled-coil 2 (CC2) domain. Previous work indicated that TBK1 ΔCC2 was signaling competent upon overexpression of TBK1. However, it was also shown that mutation of residues L693 and K694, which are located in the CC2 domain, abolishes IFN-β mRNA induction upon polyI:C transfection and VSV infection [50]. L^pro^ cleaves TBK1 at these residues. Yet, we observed that the N-terminal cleavage product restored RLR signaling in TBK1 k.o. cells upon poly(I:C) stimulation. Whether this implies that the L^pro^-mediated cleavage of TBK1 does not contribute to the viral strategy to suppress RLR signaling and IFN-β gene transcription remains unknown. Unfortunately, it is very difficult to investigate the effect of TBK1 cleavage by L^pro^ on IFN-β gene transcription in infected cells, as L^pro^ also cleaves other RLR signaling proteins, i.e. MAVS (this study) and LGP2 ([51], see below), and the transcription factor NF-κB. Dissection of the effect of TBK1 cleavage on IFN-β gene transcription from the effect of MAVS, LGP2 and NF-κB cleavage would require the identification of all L^pro^ cleavage sites in these known targets followed by the generation of cells with cleavage-resistant versions of these proteins. It remains a question whether such an approach will yield conclusive answers as other RLR signaling proteins may be targeted by L^pro^, which have not yet been identified. Hence, the relative importance of the L^pro^-mediated cleavage of TBK1 for the viral suppression of IFN-β gene transcription remains unknown.

Apart from its role in RLR signaling, TBK1 has been suggested to be involved in autophagosome maturation. TBK1 was identified as a factor in the autophagosomal clearance of herpes simplex virus 1 and mycobacteria [52,53]. A recent report showed that TBK1 phosphorylates lipidated LC-3 to prevent premature removal of LC3 from autophagosomal membranes by ATG4, thereby facilitating a unidirectional flow from the autophagosome to the lysosome [54]. Many picornaviruses hijack autophagic pathways to generate sites for viral RNA replication and to facilitate non-lytic release of virions [55–61] Possibly, cleavage of TBK1 by L^pro^ facilitates the use of autophagy to aid viral infection and propagation.

L^pro^ also impacts MAVS integrity. A distinct MAVS cleavage product was observed upon infection with EMCV-L^pro^. Remarkably, no MAVS cleavage product was observed in FMDV-infected cells, although MAVS levels progressively declined over time. The reason for this difference is unknown, but may be related to differences in human and porcine cells. Nevertheless, our data indicate that the integrity of MAVS is affected by L^pro^ in both cell types, suggesting this RLR signaling protein is targeted by FMDV to affect IFN induction. Of note, MAVS is known to localize to the peroxisomes and mitochondrion associated membranes of the ER [62,63], in addition to its default localization on mitochondria. It remains to be established whether L^pro^ targets all forms of MAVS or specifically targets MAVS at one of these locations. TBK1 and MAVS are not the only RLR signaling proteins that are targeted by L^pro^. It was previously reported that overexpression of L^pro^ induces the degradation of IRF3 and IRF7 [37], and that the p65 subunit of NF-κB is degraded in FMDV-infected cells [35]. We also observed degradation of NF-κB p65 in EMCV-L^pro^ infected-cells but we did not observe degradation of IRF3. Notably, degradation of IRF3 was also not observed in FMDV-infected cells. Whether IRF3 degradation is restricted to certain cell types or conditions, or merely is an artefact due to overexpression remains unknown.

It is remarkable that L^pro^, comprising just 173 amino acids, can carry out several specific proteolytic activities on both the viral and cellular substrates as summarized in Figs 4 and 10. Previous work documented areas of the protease that are required for polyprotein processing [41,49]. These residues included L143 which is part of the P2 pocket that can interact with leucine residues in the substrate at the P2 position. In this work, L143 was identified as being involved in TBK1 and MAVS cleavage; however, its replacement by alanine affected neither the activity on eIF4G nor the deISGylase or DUB activities. Surprisingly, mutation of two residues of the SAP domain, (I83 and L86) also affected TBK1 and MAVS cleavage, even though they are separated by 20 Å (measured between the respective C_α_ atoms) with helix α4 lying between them. Nevertheless, it cannot be excluded that the SAP mutations cause some destabilization of L^pro^, thereby explaining this mutant’s defect in proteolytic activities. Further structural work will be required to understand how L^pro^ interacts with TBK1 and MAVS.

While this work was in progress, it was reported that LGP2, a factor that is essential for MDA5 activation, is cleaved by L^pro^ [51]. The mutations in L^pro^ that impaired the reduction of IFN-β mRNA levels (L143A and mutations in the SAP domain) displayed an overall defect in the cleavage and/or degradation of each of the RLR signaling proteins we studied (i.e. MAVS, TBK1 and NF-κB p65). We anticipate that the cleavage of LGP2 is also likely impaired by introduction of these mutations. Our data suggest that expression of L^pro^ results in cleavage and/or degradation of multiple RLR signaling proteins (MAVS, TBK1, NF-κB p65, and most likely LGP2). The relative contribution of each cleavage event to the reduction in IFN-β gene transcription remains unknown. A search for other substrates of L^pro^ is of importance to further our understanding of the role and mechanism of how L^pro^ reduces IFN-α/β induction. Possibly, such a search may identify RLR signaling proteins that are cleaved earlier than the ones identified so far and may thereby have an influence on the early induction of type I IFN in infected cells.

The L^pro^ mutants that are defective in either the deISGylase/DUB activity or the cleavage/degradation of RLR signaling proteins allowed us to study which ability is needed to suppress RLR signaling. Mutation L143A, which rendered L^pro^ unable to reduce IFN-β mRNA levels, impaired the cleavage of RLR signaling molecules, but had no effect on the deISGylase or DUB activity of L^pro^. Meanwhile, mutations L92A and L102A resulted in the opposite phenotypic effect; RLR signaling proteins were cleaved with similar efficiency as wt L^pro^, but the deISGylase activity was significantly reduced by these mutations [40]. Yet, these L^pro^ mutants still reduced IFN-β mRNA levels. Collectively, these data indicate that the activity of L^pro^ to cleave/degrade RLR signaling proteins, not its deISGylase/DUB activity, is important for reduction of IFN-β induction. Medina *et*. *al*. have just reported that impairment of the deISGylation activity of L^pro^ causes viral attenuation *in vitro* and *in vivo* [64]. In support of our hypothesis, the mutations introduced by Medina *et*. *al*. did not affect IFN or ISG mRNA expression levels [64].

It was previously suggested that the DUB activity of L^pro^ is important for the suppression of RLR signaling [39], which contrasts our findings. Importantly, most experiments by Wang *et*. *al*. relied on overexpression of L^pro^, ubiquitin, and several targets proteins (i.e. RIG-I, TBK1, TRAF3 and TRAF6). A recent study showed that L^pro^ should be predominantly regarded as a deISGylase rather than a DUB, as biochemical evidence showed that L^pro^ has a 1000-fold higher activity towards ISG15 than ubiquitin [40]. Given the weak DUB activity of L^pro^ *in vitro*, it remains to be established whether L^pro^ genuinely acts as a DUB in FMDV-infected cells under physiological conditions (i.e. without overexpression of components of the ubiquitination system or known ubiquitination target proteins). Previously, we found no differences in the levels of ubiquitinated proteins in cells infected with EMCV expressing wt L^pro^ or L^pro^ C51A, whereas the levels of ISGylated protein were decreased in cells infected with EMCV-L^pro^ [40], suggesting that L^pro^ predominantly acts as a deISGylase in infected cells.

It is well established that certain viruses (i.a. adenoviruses, herpesviruses and nidoviruses) rely on viral proteases with DUB and deISGylase activity to suppress the induction of IFN-α/β [9]. FMDV L^pro^ is a papain-like protease and thus L^pro^ is best compared to other virally encoded papain-like cysteine proteases that suppress IFN-α/β gene transcription. Members of the order Nidovirales (i.e. coronaviruses and arteriviruses) encode one or two papain-like cysteine protease (PLP), referred to as PL^pro^, or PLP1 and PLP2 when the virus encodes two PLPs. In addition to cleaving the viral polyprotein, PL^pro^ and the equivalent PLP2 have acquired DUB and deISGylase activity [65–73]. Structure-guided mutagenesis of PL^pro^ of MERS-CoV and PLP2 of equine arterivirus (EAV) allowed the DUB activity to be separated from the proteolytic activity portrayed towards the viral polyprotein [72,73]. This uncoupling of these two different proteolytic activities indicated that the DUB activity of PL^pro^/PLP2 contributes to the suppression of IFN-α/β transcription [72,73]. Unfortunately, it has not been determined whether PL^pro^/PLP2 cleaves RLR signaling proteins and thus it is unclear what other proteolytic activities could be affected by the mutations that were introduced. Notably, the cleavage site of nidovirus PL^pro^/PLP2 and FMDV L^pro^ in ubiquitin and ISG15 is different. While SARS-CoV PL^pro^ breaks the iso-peptide bond between ubiquitin or ISG15 and the target protein [65,66], FMDV L^pro^ is a non-canonical deISGylase that targets a peptide bond in ISG15 itself, resulting in a diglycylated-lysine in the target protein [40]. In conclusion, nidovirus PL^pro^/PLP2 and FDMV L^pro^ are both papain-like proteases, but they likely have evolved different strategies to suppress IFN-α/β gene transcription.

FMDV L^pro^ and enterovirus 2A^pro^ are structurally different enzymes that share many functions; both cleave translation initiation factor eIF4G [34,74,75], both reduce IFN-α/β gene transcription [27,47,76,77], both have been implicated in the suppression of SG formation [77–79] and both have been suggested to rescue viral translation from the inhibitory effects of p-eIF2 [80,81]. Importantly, L^pro^ and 2A^pro^ both cleave several RLR signaling proteins, but the only overlapping RLR protein is MAVS [27,76]. Although a causal relationship between cleavage of RLR proteins and suppression of IFN-α/β transcription remains to be established for both proteases, the convergence on the cleavage of MAVS is noteworthy. In the absence of sequence homology, no evolutionary basis for the functional similarities between the two picornavirus proteases can be determined. Possibly, the extensive similarities between FMDV L^pro^ and enterovirus 2A^pro^, both picornavirus proteases, is illustrative of the urgency for picornaviruses to suppress these particular antiviral host responses.

Our data suggests that the deISGylase activity of L^pro^ is not critically needed to suppress RLR signaling, but rather its role should be sought in the broader antiviral activities of ISG15 (reviewed in [10,11]). It should be noted that our experiments were performed in naïve cells at high multiplicity of infection. Expression of the ISGylation machinery as well as ISG15 itself are boosted by IFN-α/β and therefore we cannot formally exclude a role for the DUB and/or deISGylase activity of L^pro^ in suppressing RLR signaling under different conditions (e.g. in IFN-primed cells). ISG15 has many functions, both intracellular and extracellular. Intracellular ISG15 can act both pro-inflammatory and immunomodulatory, either via ISGylation of target proteins or as free ISG15. Moreover, ISG15 can be secreted to act as a cytokine [25,26]. ISG15 also plays a role in damage repair after clearing viral infection [82] and can regulate cellular processes such as autophagy and metabolism [24,83–85]. How the deISGylase activity of L^pro^ contributes to efficient *in vivo* infection, remains to be established.

## Materials and methods

### Cells and viruses

HeLa R19, HeLa R19 TBK1 k.o. and Hek293T cells were maintained in Dulbecco’s Modified Eagle’s Medium (DMEM) supplemented with 10% FCS (V/V). HeLa OHIO cells were maintained in DMEM supplemented with 10% FCS (V/V) and 1% penicillin/streptomycin. LFPK αv*β*6 cells [86] were obtained from the Foreign Animal Disease Diagnostic Laboratory (FADDL) at the PIADC. These cells were maintained in minimal essential medium (MEM) supplemented with 10% FCS (V/V) and 1% antibiotics and non-essential amino acids. BHK-21 cells used for FMDV propagation were maintained in MEM supplemented with 10% FCS (V/V), 10% tryptose phosphate broth, 1% antibiotics and non-essential amino acids. HeLa R19 TBK1 k.o. cells were generated via CRISPR/cas9 methodology using a pCRISPR plasmid, as described previously [87]. The used gRNA sequences are 5’-GCTACTGCAAATGTCTTTCG-3’ and 5’-GAGGAAAACAGATTGGTT-3’. FMDV A12-WT (wild type) was generated from the full-length serotype A12 infectious clone, pRMC35 [88] and A12-LLV2 (leaderless virus) was derived from the infectious clone lacking the Lb coding region, pRM-LLV2 [89]. Viruses were propagated in BHK-21 and concentrated by polyethylene glycol precipitation, titrated on BHK-21 cells, and stored at -70°C. ERAV (NM-11/67 strain) (gift from D. Rowlands and T. Tuthill [90]) was obtained after passage on HeLa R19 cells and subsequently concentrated by ultracentrifugation through a 30% sucrose cushion at 140,000xg for 16 hours in a SW32Ti rotor and stored at -80°C. Recombinant EMCVs were generated by cloning the genes of interest into the XhoI/NotI restriction sites from the pM16.1-VFETQG-Zn infectious clone that was described previously [45]. EMCV-L^pro^ viruses were recovered by transfection of run-off RNA transcripts into BHK-21 cells. Upon total CPE, viruses were concentrated by ultracentrifugation (as described for ERAV) and stored at -80°C.

### Antibodies

The following antibodies were used for Western blot staining procedures: αFMDV VP1 (rabbit polyclonal Abmade at PIADC), αEMCV capsid (gift from Ann Palmenberg), αL^pro^ (gift from Ewald Beck and Tim Skern), αMAVS (Enzo life science ALX-210-929), αTBK1 (Cell signaling 3504), αIRF3 (Santa cruz sc-9082), αNF-κB-p65/RelA (Santa Cruz Biotechnology SC-8008), αeIF4G (Bethyl laboratories A300-502A), αG3BP1 (BD biosciences clone 23/G3BP), αPARP (Roche Diagnostics #11835238001), αFLAG (Sigma M2), αGFP (Invitrogen OSE00003G), αHis (GE Healthcare, 27-4710-01), αMyc (Clone 4A6, Millipore), αHA (Abcam ab130275) and αtubulin (Sigma DM1A). Respective IRdye680 or IRdye800 conjugated secondary antibodies (LiCOR) or HRP-conjugated secondary antibodies were used for detection.

### RT-PCR analysis

HeLa R19 cells were seeded in 24-wells plates and the next day infected with the indicated viruses at MOI 10 or transfected with the indicated plasmids or vRNA. Plasmids were transfected using Fugene6 (Promega) and vRNA was transfected using Lipofectamine 2000 (Invitrogen), both according to the manufacturer’s instructions. Preparation of viral dsRNA and the pcDNA-GFP-MAVS construct have been described previously [1,44]. pEGFP-IRF3 [D5] construct was a kind gift from John Hiscott [91]. At the indicated time points cells were lysed and cellular RNA was isolated using total RNA isolation kit (Machery-Nagel) according to manufacturer’s instructions. Reverse transcription was set up using TaqMan Reverse Transcription Reagents (Applied Biosystems) before performing qPCR analysis with SYBR green (Roche) as described previously [92].

### Western Blot analysis of transfected cells

The pIRES-EGFP-FMDV L^pro^ plasmid was described previously [45]. The pcDNA-FLAG-TBK1 plasmid was a gift from John Hiscott [93] and the pEF-FLAG-RIG-I was a gift from Takashi Fujita [94]. HA-ubiquitin was expressed from a pCMV5 plasmid. Hek293T cells were seeded in 6-well plates and the next day transfected with 1.5 μg of total plasmid using Fugene6 (Promega) according to manufacturer’s instructions. 16h posttransfection cells were lysed 100 μl lysisbuffer (100 mM Tris pH 8.0, 1 mM EDTA, 50 mM NaCl, 1% NP40, protease inhibitor mix (Roche)). Post nuclear lysate was obtained by centrifugation at 15000xg at 4°C for 15 min. The amount of total protein in the lysates was determined using BCA assay (ThermoFisher) and 100 μg of protein was resolved using reducing sodiumdodecyl sulfate-polyacrylamide gel electrophoresis (SDS-PAGE) and transferred to 0.2 μm nitrocellulose membranes by wet electrophoretic transfer. Membranes were incubated 1h in blocking buffer (PBS + 0.1% Tween 20 + 2% BSA) and successively incubated overnight with primary antibodies diluted in blocking buffer and then for 30 min with respective secondary antibodies diluted in blocking buffer. Between and after the incubations, the membranes were washed three times with PBS+0.1% Tween-20. Finally, membranes were washed once with PBS and scanned using an Odyssey Imager (Li-COR).

### Western Blot analysis of infected cells

HeLa R19 cells were seeded in 10 cm dishes and infected the next day with the indicated viruses at MOI 10. At the indicated time points cells were released using trypsin, washed once in PBS and lysed in 100 μl lysis buffer (100 mM Tris pH 8.0, 1 mM EDTA, 50 mM NaCl, 1% NP40, protease inhibitor mix (Roche)). Subsequent steps are identical as described for transfected cells. For the analysis of FMDV-infected LFBK αv*β*6 cultures, cells were lysed in lysis buffer (0.5% NP-40 substitute, 50mM Tris pH 7.5, 150mM NaCl, 1mM EDTA). Lysates were incubated at 4°C for 10 min and cellular debris was collected by centrifugation at 10,000xg for 15 min at 4°C. 40 ng of protein was resolved by SDS-PAGE, transferred by Western blot and secondary antibodies conjugated with horseradish peroxidase (Pierce) were used for detection of proteins. Following incubation with appropriate primary and secondary antibodies, protein bands were visualized using SuperSignal West Dura Extended Duration Substrate (ThermoScientific, Rockford, IL, USA) according to the manufacturer’s directions.

### In vitro TBK1 cleavage

sL^pro^ was expressed and purified as reported previously [95]. 275 ng of His-hTBK1 (Millipore) was incubated with 0–3 μg sL^pro^ for 2 h at 30°C in a HEPES buffer (20 mM HEPES pH 7.4, 150 mM KCl, 1 mM EDTA) before the reaction mixture was dissolved on SDS-PAGE. Proteins were transferred to nitrocellulose and Western blot staining for the his-tag was performed. Myc-mTBK1 and Myc-mTBK1_692AAA694_ were transiently expressed in HeLa OHIO cells from plasmid pCS2-6Myc-mTBK1, a gift from T. Decker. 20 μg myc-tagged mTBK1 containing cell lysate was incubated with 2 μg sL^pro^ for 2 h at 30°C in a HEPES buffer before resolving the reaction mixture on SDS-PAGE, transferring the protein to nitrocellulose membrane and performing Western blot staining for Myc.

### In vitro DUB and deISGylase assays

Ubiquitin/ISG15-TAMRA assays were performed according to [96]. Di-ubiquitin *in vitro* cleavage assays were performed as described previously [97].

## Supporting information

S1 Fig N-terminal TBK1 cleavage fragment can facilitate RLR signaling. **(A)** HeLa R19 TBK1 k.o. cells were generated using CRISPR/cas9 technology. Wt and TBK1 k.o. cells were lysed and lysates subjected to Western Blot analysis for TBK1 and tubulin. **(B)** IFN-β induction upon various triggers of RLR signaling was compared in wt and TBK1 k.o. cells. Cells were infected with EMCV-L^Zn^ virus at MOI 10, transfected with 20 ng vRNA, or transfected with 1 μg of plasmid expressing MAVS or IRF3. Cells were lysed at 8 h pi or transfection. Total RNA was isolated and used for RT-qPCR analysis for IFN-β and actin mRNA. The IFN-β levels are depicted as a fold induction compared to levels in mock-treated cells, after correction for actin mRNA levels. Error bars depict the SD. **(C)** HeLa R19 TBK1 k.o. cells were transfected with 2 μg plasmid expressing full-length or truncated TBK1 (TBK1 Δ35aa). TBK1 Δ35aa is representative for the L^pro^-generated N-terminal cleavage product. Cells were lysed and lysates subjected to Western Blot analysis for TBK1 and tubulin. **(D)** TBK1 k.o. cells were reconstituted with full-length TBK1 as described for (C) and subsequently transfected with 100 ng poly(I:C). Cells were lysed at 8 h post transfection of poly(I:C). Total RNA was isolated and used for RT-qPCR analysis for IFN-β and actin mRNA. The IFN-β levels are depicted as a fold induction compared to levels in mock-treated cells, after correction for actin mRNA levels. Error bars depict the SD. **(E)** TBK1 k.o. cells were reconstituted with full-length or truncated TBK1 (TBK1 Δ35aa) as described for (C). Subsequent steps as described for (D). Error bars depict the SD.
(TIF)
