# Supplementary figures and images for: Dissecting distinct proteolytic activities of FMDV Lpro implicates cleavage and degradation of RLR signaling proteins, not its deISGylase/DUB activity, in type I interferon suppression
Source: PLoS Pathog. 2020 Jul 15;16(7):e1008702. doi: 10.1371/journal.ppat.1008702 (PMC7384677; doi:10.1371/journal.ppat.1008702)

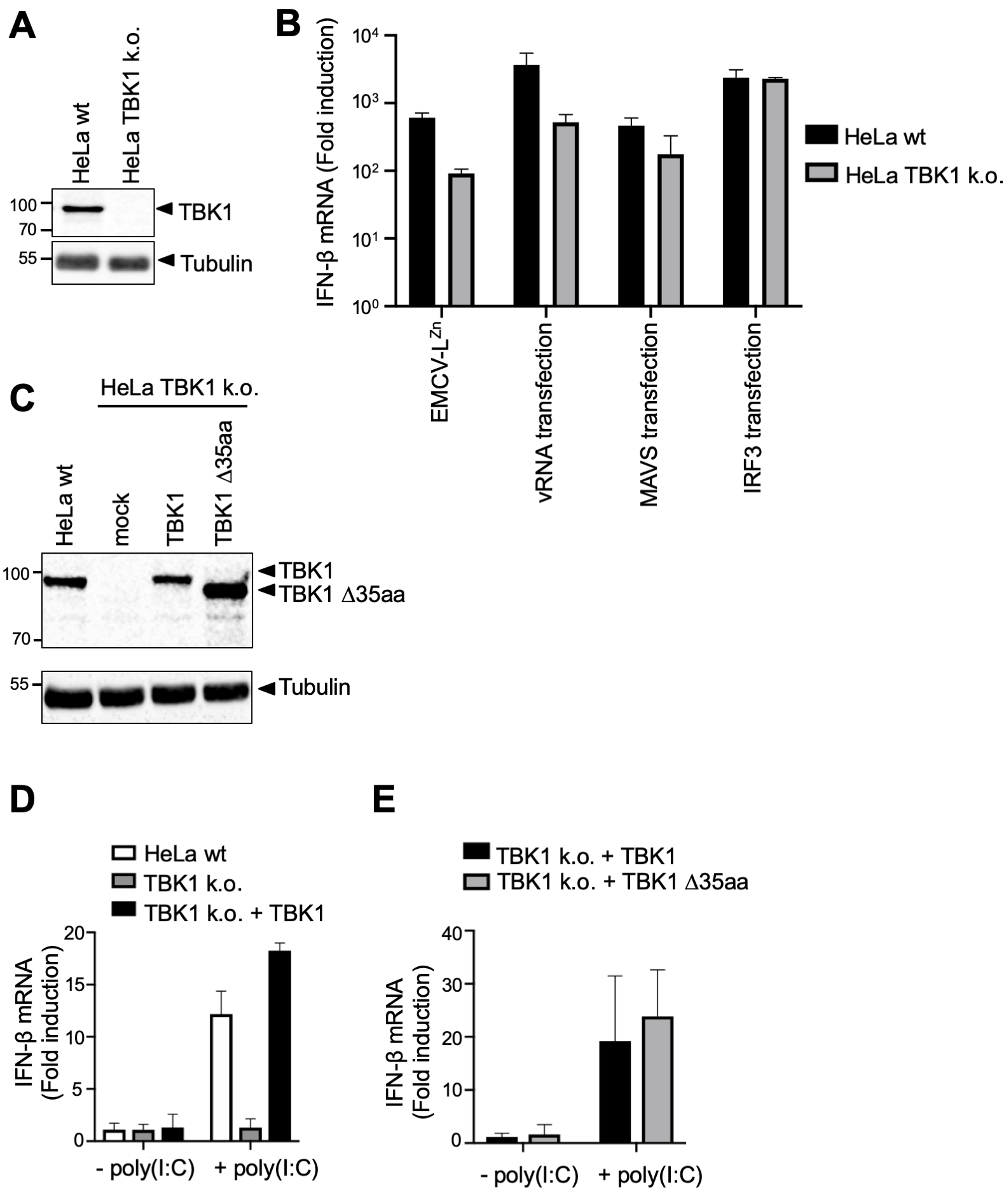

Supplement: S1 Fig — (A) HeLa R19 TBK1 k.o. cells were generated using CRISPR/cas9 technology. Wt and TBK1 k.o. cells were lysed and lysates subjected to Western Blot analysis for TBK1 and tubulin. (B) IFN-β induction upon various triggers of RLR signaling was compared in wt and TBK1 k.o. cells. Cells were infected with EMCV-LZn virus at MOI 10, transfected with 20 ng vRNA, or transfected with 1 μg of plasmid expressing MAVS or IRF3. Cells were lysed at 8 h pi or transfection. Total RNA was isolated and used for RT-qPCR analysis for IFN-β and actin mRNA. The IFN-β levels are depicted as a fold induction compared to levels in mock-treated cells, after correction for actin mRNA levels. Error bars depict the SD. (C) HeLa R19 TBK1 k.o. cells were transfected with 2 μg plasmid expressing full-length or truncated TBK1 (TBK1 Δ35aa). TBK1 Δ35aa is representative for the Lpro-generated N-terminal cleavage product. Cells were lysed and lysates subjected to Western Blot analysis for TBK1 and tubulin. (D) TBK1 k.o. cells were reconstituted with full-length TBK1 as described for (C) and subsequently transfected with 100 ng poly(I:C). Cells were lysed at 8 h post transfection of poly(I:C). Total RNA was isolated and used for RT-qPCR analysis for IFN-β and actin mRNA. The IFN-β levels are depicted as a fold induction compared to levels in mock-treated cells, after correction for actin mRNA levels. Error bars depict the SD. (E) TBK1 k.o. cells were reconstituted with full-length or truncated TBK1 (TBK1 Δ35aa) as described for (C). Subsequent steps as described for (D). Error bars depict the SD. (TIF) [file ppat.1008702.s001.tif]
